# Preparation of Poly (dl-Lactide-co-Glycolide) Nanoparticles Encapsulated with Periglaucine A and Betulinic Acid for In Vitro Anti-*Acanthamoeba* and Cytotoxicity Activities

**DOI:** 10.3390/pathogens7030062

**Published:** 2018-07-16

**Authors:** Tooba Mahboob, Muhammad Nawaz, Tan Tian-Chye, Chandramathi Samudi, Christophe Wiart, Veeranoot Nissapatorn

**Affiliations:** 1 Department of Parasitology, Faculty of Medicine, University of Malaya, Kuala Lumpur 50603, Malaysia; tooba666@hotmail.com (T.M.); tantianchye@um.edu.my (T.T.-C.); 2 Department of Medical Microbiology, Faculty of Medicine, University of Malaya, Kuala Lumpur 50603, Malaysia; chandramathi@um.edu.my; 3 Department of Nano-Medicine Research, Institute for Research and Medical Consultations (IRMC), Imam Abdulrahman Bin Faisal University, P.O. Box 1982, Dammam 31441, Saudi Arabia; 4 School of Pharmacy, Nottingham University Malaysia Campus, Semenyih, Selangor, Kuala Lumpur 43500, Malaysia; christophe.wiart@nottingham.edu.my; 5 School of Allied Health Sciences, Walailak University, Nakhon Si Thammarat 80161, Thailand; 6 Research Excellence Center for Innovation and Health Products, Walailak University, Nakhon Si Thammarat 80161, Thailand

**Keywords:** anti-*Acanthamoeba*, betulinic acid, cytotoxicity, periglaucine A, PLGA-nanoparticles

## Abstract

Poly (dl-lactide-co-glycolide) (PLGA) microspheres were synthesized as delivery system for the natural anti-parasitic compounds, Periglaucine A (PGA) and Betulinic acid (BA). Periglaucine A and Betulinic acid were encapsulated in PLGA nanoparticles by single emulsion method with an average particle size of approximately 100–500 nm. Periglaucine A and Betulinic acid encapsulation efficiency was observed to be 90% and 35% respectively. Anti-*Acanthamoeba* property of Periglaucine A and Betulinic acid remained intact after encapsulation. PGA-PLGA and BA-PLGA nanoparticles demonstrated inhibition in viability of *Acanthamoeba triangularis* trophozoites by 74.9%, 59.9%, 49.9% and 71.2%, 52.2%, 88% respectively at concentration of 100 µg/mL, 50 µg/mL and 25 µg/mL. Cytotoxicity of PGA-PLGA and BA-PLGA nanoparticles has been evaluated against lung epithelial cell line and showed dose dependent cytotoxicity value of IC_50_ 2 µg/mL and 20 µg/mL respectively. Futher, increased viability was observed in lung epithelial cell line in higher doses of synthesized polymeric nanoparticles. Results indicate that poly (dl-lactide-co-glycolide) (PLGA) nanoparticles could be exploratory delivery systems for natural products to improve their therapeutic efficacy.

## 1. Introduction

*Acanthamoeba* spp. are amphizoic and opportunistic pathogens, which have been isolated from a range of environmental conditions all over the world including soil, dust, air, tap water, drinking water, swimming pools and air-conditioning units as well as from various animals and the mucosa of healthy humans [1]. There are two life stages of *Acanthamoeba*: an infective trophozoite stage characterized by the presence of acanthapodia and prominent vacuoles and a smaller, metabolically-inactive cyst stage characterized by the presence of an endocyst and an ectocyst that morphologically resemble triangles, stars, cubes or circles. Trophozoites feed on bacteria, algae or yeast or grow axenically on nutrients [1]. Encystment occurs when conditions become unfavorable, such as during lack of nutrients or desiccation and cysts are highly resistant to temperature and pH changes [2].

*Acanthamoeba* is the causative agent of granulomatous amebic encephalitis (GAE), a fatal disease most commonly infecting immunocompromised people and amebic keratitis, which is a painful inflammation of the cornea that can lead to vision loss and is associated with use of contact lenses. *Acanthamoeba* infections have also manifested as pneumonitis, nasopharyngeal infections and cutaneous infections and are also capable of disseminating to other areas of the body [1,3]. Immunological memory does not develop against *Acanthamoeba*, making re-infection possible [1]. Current treatments involve combinations of anti-microbial agents including polyhexamethylene biguanide, propamidine isethionate, neomycin and chlorhexidine [4]. While these therapies are often effective against the trophozoite stage, treatments with potent anti-cyst effects have yet to be established, resulting in prolonged infection in patients.

Recent studies have increasingly been investigating and validating the antibiotic effects of phytochemicals derived from medicinal plants that have traditionally been used to fight parasitic infection. This includes the Asian plant *Pericampylus glaucus*, from which periglaucine A—an alkaloid—and terpenes such as betulinic acid have been derived [5,6]. These compounds were previously found to demonstrate anti-tumor activity, anti-viral activity against HBV and HIV and anti-inflammatory activity through inhibition of COX enzymes [6,7,8,9]. As was studied earlier, these compounds have shown significant activity against *Acanthmaoeba triangularis* [10]. However, this study had been proposed for deeper evaluations such as comparing biological activities of these pure compounds and studies of encapsulation within the polymeric nanoparticles. In addition, the purpose of nanoencapsulation of pure compounds includes effective and efficient penetration in the blood brain barriers, which is the targeted site in *Acanthamoeba* infections such as encephalitis. Most of the available commercial drugs administered with high concentration in order to maximize concentration of drug at targeted site usually result in high cytotoxicity and other side effects. Moreover, the solubility of terpenes as Betulinic acid contributes to a major problem in drug design. Nanoencapsulation can help to improve the solubility of compounds as well as efficacy. Moreover, a recent study demonstrated that gold conjugated chlorhexidine nanoparticles had significantly enhanced amoebicidal and amoebistatic effects [11]. The other study observed improved efficacy of antiamoebic drugs by silver nanoparticle conjugation [12].

When conventional therapies fail, the advancing field of nanotechnology is increasingly being used to improve or replace them. Encapsulation of drugs or biomolecules in nanoparticles for delivery has been shown to have numerous pharmacological and bioengineering applications, such as the delivery of genes, anti-parasitic drugs, proteins, anti-viral drugs and chemotherapeutics, as well as use in ligament reconstruction, surgical dressings, imaging, dental and fracture repairs [13,14]. Nanoparticles are about 100 nm in diameter and can carry their therapeutic agent by encapsulation or surface conjugation [15]. Degradation time can vary from months to years, depending on their production and composition. In general, degradation time increases with hydrophobicity and molecular weight [15].

Many parameters of nanoparticle production influence their size, degradation rate, encapsulation efficiency and biocompatibility, making them highly versatile. Therefore, materials and methods must be optimized and tailored to their cargo and targets. This includes the type and amount of organic solvents and surfactants used in the emulsification method and the co-polymer ratio. Nanoparticles can allow for sustained release of molecules, thereby reducing dosage frequency. This augments cytotoxic effects, making them safer by allowing lower minimum doses [9]. Surface modification can also allow for tissue-specific targeting, increasing the drug’s local bioavailability and reducing systemic side effects [9,16]. Surface modification can also be achieved to increase the particle’s hydrophilicity and avoid their clearance from the blood by the reticuloendothelial system [15]. All of above mentioned parameters were taken into consideration while synthesizing betulinic acid and periglaucine A nanoparticles to reduce dosage concentration and enhancing hydrophilicity.

Poly (dl-lactide-co-glycolide), or PLGA, is a co-polymer commonly used in nanotechnology and is approved by the FDA for drug delivery. Hydrolytic cleavage of PLGA’s ester bonds results in lactide and glycolide monomers, which are further metabolized by the citric acid cycle. As such, PLGA is biodegradable and biocompatible. Due to the submicron size of PLGA nanoparticles, they are easily absorbed and extravasated through capillaries, allowing for access into less-permeable tissue [13].

Nanoparticles can be formed in a variety of ways, including emulsion evaporation, emulsion diffusion, salting out, nanoprecipitation and solvent displacement. For PLGA particles, the most common methods for formation are single emulsion evaporation (better for hydrophobic cargo) and double emulsion evaporation (better for hydrophilic cargo) [15]. In the present proof-of-concept study, PLGA nanoparticles encapsulating periglaucine A and betulinic acid were prepared by the single emulsion-evaporation method and their amoebicidal potential and cytotoxic effects were investigated.

## 2. Results

### 2.1. Preparation and Characterization of PGA-PLGA and BA-PLGA Nanoparticles

**Figure 1:**
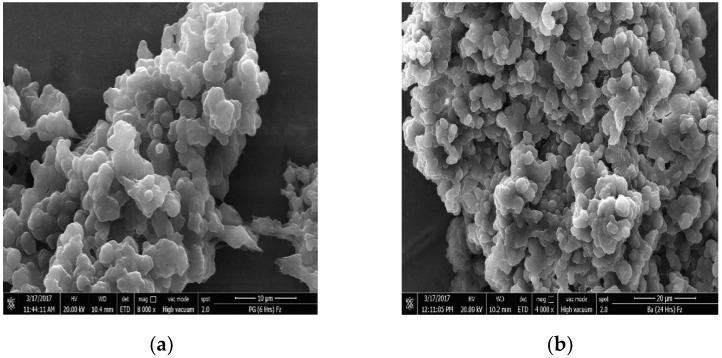
SEM images of synthesized nanoparticles: (**a**) SEM image of PGA-PLGA nanoparticles; (**b**) SEM image of BA-PLGA nanoparticles.

**Figure 2:**
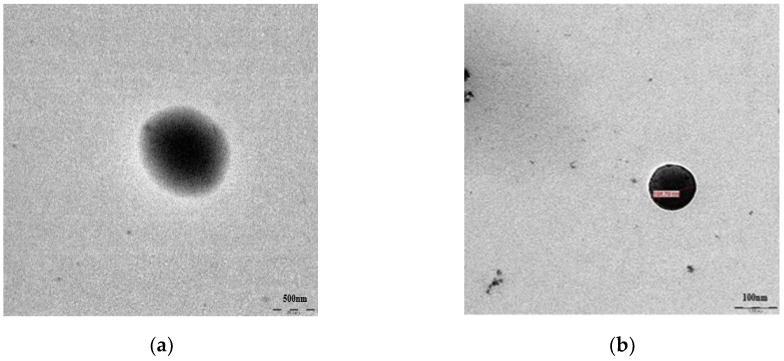
TEM images of synthesized nanoparticles: (**a**) TEM image of PGA-PLGA nanoparticles; (**b**) TEM image of BA-PLGA nanoparticles.

The PLGA nanoparticles encapsulated with periglaucine A and betulinic acid were successfully synthesized by single emulsion solvent evaporation. Different batches of nanoparticles were observed for consistency. The synthesized PGA-PLGA and BA-PLGA nanoparticles were completely soluble in water and showed absorbance at the lambda max of 630 nm and 210 nm. The prepared PGA-PLGA nanoparticles were monodispersed with approximate size 500 nm and average size of synthesized BA-PLGA nanoparticles were approximately 100 nm by observing under scanning electron microscopy and transmission electron microscopy. The particle sizes of the prepared nanoparticles differ from each other, it may be due to different chemical nature of molecules and other methods of synthesizing nanoparticles could be optimized. The smooth surface of synthesized nanoparticles implied complete coating by PLGA. Scanning electron microscopy and transmission electron microscopy were performed to observe the surface morphology of synthesized nanoparticles at the Electron Microscopy (EM) unit, Faculty of Medicine, University of Malaya. The morphology of PGA-PLGA and BA-PLGA were found to be spherical in shape with approximately 100–500 nm size (Figure 1 and Figure 2).

### 2.2. Encapsulation Efficiency of Periglaucine A and Betulinic acid in PLGA

The compound content of nanoparticles was determined by a spectrophotometer (SpectraMax M3, Multi-Mode Microplate reader, Molecular Devices, Washington, DC, USA). The absorbance of free periglaucine A was measured at wavelength 630 nm and betulinic acid was measured at 210 nm. The concentration of periglaucine A and betulinic acid were calculated from a standard curve, prepared by measuring the absorbance of known concentrations of free active compound. Periglaucine A was successfully found to be 91.20% encapsulated inside PLGA nanoparticles whereas betulinic acid was 35.90%. Mostly, encapsulation efficiency depends upon the hydrophobic interaction between the active compound and polymer, less hydrophobic compounds exhibit higher entrapped efficiencies. In this study, the lower encapsulation efficiency of betulinic acid can be relative to its hydrophobic nature while higher encapsulation efficiency (above 90%) of periglaucine A can be related to its hydrophilic characteristics.

### 2.3. In Vitro Release Study

**Figure 3:**
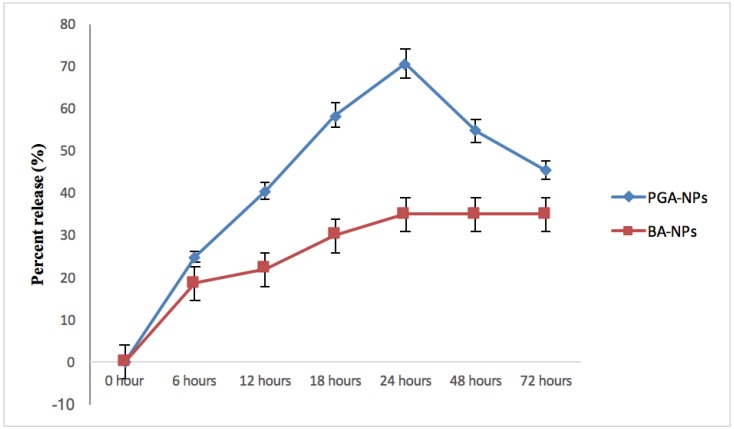
In vitro release of periglaucine A and betulinic acid.

We evaluated the periglaucine A and betulinic acid release from PLGA nanoparticles as a function of time. The periglaucine A and betulinic acid in vitro release from PLGA nanoparticles was studied in Phosphate buffer saline, PBS (pH 7.5) at interval of 6, 12, 18, 24, 48 and 72 h. It was observed that after 24 h, periglaucine A release was 70.6% and betulinic acid was 30%. However, after 48 and 72 h, the percent released of periglaucine A was decreased to 54.8% and 45.4% respectively while betulinic acid maintained 30% release after 48 and 72 h measurement (Figure 3).

### 2.4. Amoebicidal Potential of Synthesized Nanoparticles

The trophocidal activity of PGA-PLGA and BA-PLGA NPs were found to be remarkably enhanced in comparison with chlorhexidine at 25 µg/mL. At a concentration of 100 µg/mL, 50 µg/mL and 25 µg/mL, PGA-PLGA NPs inhibited cell viability by 74.9%, 59.9% and 49.9% after 72 h against the trophozoites stage (Table 1) while having a 42.1% inhibition against the cyst stage at 100 µg/mL. At 100 µg/mL, PGA-PLGA nanoparticles inhibited trophozoites viability by 57.4% after 24 h. Whereas PGA-PLGA nanoparticles showed the inhibition of cyst viability by 29.1% after 24 h. This inhibition was increased to 32.5% after 48 h and 42.1% after 72 h. BA-PLGA nanoparticles at concentration 100 µg/mL, showed inhibition of trophozoites by 71.3% after 72 h which was found to be superior in comparison to chlorhexidine (Table 2). The viability of cysts was inhibited to 47.6% by BA-PLGA nanoparticles at 50 µg/mL (Table 2). PGA-PLGA nanoparticles demonstrated time as well as dose dependent activity against the cyst stage of *Acanthamoeba triangularis*.

### 2.5. Cytotoxicity

PGA-PLGA and BA-PLGA nanoparticles were evaluated against MRC-5 cell line. Cytotoxicity of PGA-PLGA nanoparticles was found to be superior in comparison with the parent compound itself, which indicates the cytotoxic effects of synthesized PGA-PLGA nanoparticles. Similarly, BA-PLGA nanoparticles were found to be cytotoxic to MRC-5 cell line with IC_50_ 20 in comparison to betulinic acid without encapsulation (Table 3). As the dose of PGA-PLGA NPs and BA-PLGA NPs were increased, the viability of lung epithelial cell was also found to be increased. However, beneficial and potential harmful effects of PGA-PLGA NPs and BA-PLGA NPs must be weighed in future studies.

### 2.6. Mode of Action of Synthesized Nanoparticles

**Figure 4:**
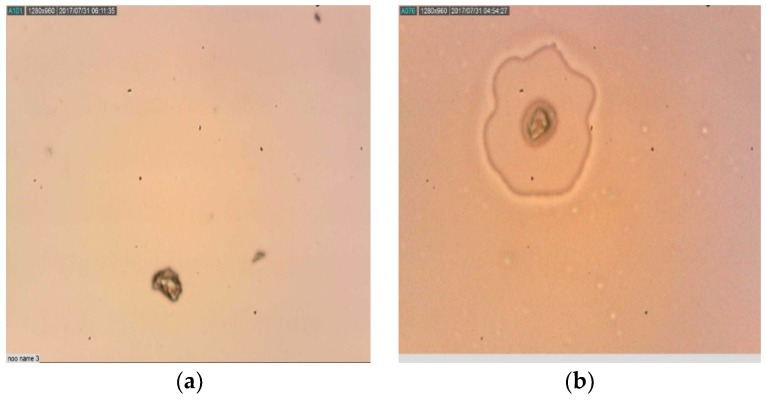
(LiM × 100) *Acanthamoeba triangularis* trophozoites treated with synthesized nanoparticles with no sign of apoptosis (No brown precipitate): (**a**) Trophozoites treated with PGA-PLGA nanoparticles; (**b**) Trophozoites treated with BA-PLGA nanoparticles; (LiM = Light microscopy).

DNA fragmentation was not observed in PGA-PLGA and BA-PLGA NPs using the TUNEL assay as no brown precipitate was observed inside cells (Figure 4). This system relies on the binding of terminal deoxynucleotidyl transferase (TdT) at the end of DNA fragments which are developed after apoptosis fragmentation. There was no induction of apoptosis in *Acanthamoeba* trophozoites by treating PGA-PLGA and BA-PLGA nanoparticles. Though programmed cell death in *Acanthamoeba* was observed when it was infected with *Salmonella*. Relative to these observations, specific pathways and molecules involved still remain unstudied.

## 3. Discussion

Targeted delivery can result in a higher concentration of therapeutic agent at its site of action, thus simultaneously reducing both the total dose and cytotoxicity and side effects associated with the drug. The application of nanoparticles for drug targeting in vivo has attracted considerable interest in the therapy of several diseases such as cancer, genetic disorders, viral and bacterial infections in specific body sites [13,17]. Periglaucine A has been reported to possess anticancer and anti-inflammatory potential [6] whereby betulinic acid has been shown to demonstrate antitumor, anti-HIV, antimalarial, anthelminthic and antioxidant properties [7,18]. These biologically active compounds can serve as valuable candidates for future development of anti-*Acanthamoeba* agents with an adequate drug delivery system.

Entrapment efficiency is used to indicate the amount of compound entrapped into the polymeric matrix. The entrapment efficiency for periglaucine A was 91.2%. In vitro release profile of periglaucine A was also evaluated as a function of time. The release of compound depends on the nanoparticle composition [19]. Generally, the mechanisms by which active agents can be released from a delivery system are the combination of diffusion of the active agent passing through the polymer, swelling, polymer erosion and degradation [20]. Usually the degradation of PLGA is slow; hence the release mechanism of antimicrobial compounds may depend on substance diffusion and PLGA surface and bulk erosion [20]. In our release system, an average release of periglaucine A was about 70.6% and betulinic acid was 30% after 24 h. 

In the present study, we attempted to develop novel PGA-PLGA and BA-PLGA nanoparticles and examined their anti-*Acanthamoeba* activity against *A. triangularis* and showed significant trophocidal activity at lower concentrations. Whereas synthesized BA PLGA-nanoparticles and PGA-PLGA nanoparticles showed moderate cysticidal activity after 72 h. In order to observe cytotoxicity, tests were performed against lung epithelial cells line (MRC-5) PGA-PLGA NPs showed higher cytotoxicity at lower doses and BA-PLGA NPs cytotoxicity was also observed to be more cytotoxic at lower doses in comparison with bulk betulinic acid. The viability of lung epithelial cell lines was increased when the dose of synthesized PLGA nanoparticles encapsulated with periglauicne A and betulinic acid increased. Further studies on the potential and harmful effects of PLGA- nanoparticles is warranted.

The programmed cell death in unicellular parasites has been so far reported in *Lesihmania*, *Plasmodium*, *Blastocystis hominis* and *Entamoeba histolytica*. Currently, the existing literature on apoptosis in *Acanthamoeba* is very limited. A type 1 meta caspase has been reported in *Acanthamoeba*. Though its function seems to be more associated to the encystation process and with osmoregulation rather than with apoptosis. However, it is well known that not all the members of caspase family are involved in programmed cell death. In previous studies, Betulinic acid and periglaucine A have been reported to induce apoptosis in several tumor cells by modifying its mitochondrial function. The mechanism of prepared nanoparticles encapsulated with betulinic acid and periglaucine A was also observed for programmed cell death as apoptosis induction by Tunel method via Peroxidase in situ apoptosis detection kit. No fragmentation in DNA strand was observed inside cells indicating that the inhibitions of cells by nanoparticles was not due to apoptosis induction (Figure 3).

It is worth noting that infections caused by *Acanthamoeba* are increasing year after year [21]. Based on the positive results observed, it could be assumed that PGA-PLGA NPs could be able to effectively remove *Acanthamoeba* from the site of infection with the minimum dose and toxicity. Further intensive studies are certainly required to determine the effects of these NPs on different species of pathogenic *Acanthamoeba* as well as their underlying mechanisms of anti-parasitic actions, with reference to periglaucine A and betulinic acid. The present proof of concept study on PGA-PLGA and BA-PLGA NPs may serve as a baseline data for further in vitro and more clinical studies on this promising novel anti-parasitic agent.

## 4. Materials and Methods

### 4.1. Chemicals and Reagents

PLGA (Mol. wt. 76.05), PVA (Mol wt. 89,000–98,000), quercetin, chlorhexidine and MTT (3-(4,5-dimethylthiazol-2-yl)-2,5- diphenyltetrazolium bromide), DAB (Diaminobenzidine) and RPMI 1640 were purchased from Sigma Aldrich Ltd. (St. Louis, MI, USA). Fetal bovine serum (FBS) was purchased from Biosera (Kansas City, MO, USA). Trypan blue, Phosphate buffer saline (PBS), Dichloromethane (DCM), Ethanol, Paraformaldehyde and Dimethyl sulfoxide (DMSO) were purchased from IDL Scientific Ltd. (Kuala Lumpur, Malaysia). Periglaucine A, betulinic acid, dichloromethane ChemFaces Ltd. (Hubei, China). In situ apoptosis detection kit was purchased from Millipore, Merck (MA, USA).

### 4.2. Preparation of PGA loaded PLGA Nanoparticles

Loading of periglaucine A and betulinic acid onto PLGA NPs was done by single emulsion method. 0.02 g of Poly dl-lactide-co-glycolide (PLGA) and 0.005 g of each periglaucine A and betulinic acid were dissolved in 2 mL of dichloromethane (DCM) and vortexed for one minute until completely dissolved. This organic phase mixture was then emulsified by drop wise addition to 20 mL of an aqueous phase containing 0.25% PVA. The samples were emulsified for 10 min by stirring and then sonicated at 50% amplitude for 3 min by ultrasonic probe sonicator, Athena technology (Maharashtra, India). The organic solvent was evaporated by stirring, Hotplate Stirrer LMS-1003, Labtech (Washington, DC, USA) for 4 h. Control nanoparticles were prepared by the same procedure without the addition of active plant constituents. The NPs suspended in emulsion were collected by ultracentrifugation at 1000 rpm for 5 min and washed thrice with distilled water. The synthesized nanoparticles were poured into semi-stoppered glass vials with sloterred rubber closure at maximal height of 10 mm. The prepared periglaucine A-PLGA (PGA-PLGA) and betulinic acid-PLGA (BA-PLGA) nanoparticles were lyophilized in a freeze dryer, Emitech K750X, Quorum Technologies (Lewes, UK) at −53 °C for over-night, followed by first drying step at −45 °C and pressure of 400 mTorr followed by secondary drying step at 20 °C and pressure of 200 mTorr. After 24 h, dried nanoparticles were transferred into 1.5 mL Eppendorf tubes and stored at −20 °C.

### 4.3. Morphology Study of PGA-PLGA Nanoparticles

PGA-PLGA or BA-PLGA NPs morphology was assessed by scanning electron microscopy (SEM) and transmission electron microscopy (TEM). SEM was performed on a FESEM, Quanta FEG 650, FEI, Hillsboro (OR, USA). For sample preparation, 0.5 mg of PGA-PLGA and BA-PLGA nanoparticles was mixed with 0.4 mL of absolute ethanol in 1.5 mL Eppendorf tubes and sonicated in an ultrasonic bath sonicator, Sonorex RK 100H (Eichenau, Germany) for about 5 min. After sonication, 20 µL of the solution was transferred to a piece of aluminum foil and excessive solvent was removed. It was then left at room temperature for 1–1.5 min. The piece of foil was transferred to a carbon adhesive tape and incubated at 37 °C for 5 min. The sample was then coated with a mixture of gold in an argon atmosphere and viewed under SEM. Whereas for TEM PGA-PLGA and BA-PLGA NPs were suspended in ethanol at 2 mg/mL with sonication for 5 min. A sample of 5 µL was deposited on a freshly glow-discharged 300 mesh carbon coated copper TEM grid, Ted Pella Inc. (Redding, CA, USA) and allowed to adhere for 4 to 5 min. Excess solvent (ethanol) was removed with filter paper. Samples were kept in oven at 45 °C for one hour. Samples were immediately imaged using TEM, TEM Leo Libra-120, Carl Zeiss AG (Oberkochen, Germany) at an accelerating voltage of 100 kV.

### 4.4. Determination of Periglaucine A and Betulinic acid Encapsulation Efficiency

The compound content of nanoparticles was determined by a spectrophotometer, SpectraMax M3, Multi-Mode Microplate reader, Molecular Devices (Washington, DC, USA). The absorbance of free periglaucine A and betulinic acid in NPs was measured at 630 nm and 210 nm wavelength. The concentration of periglaucine A and betulinic acid were calculated from a standard curve, prepared by measuring the absorbance of known concentrations of free active compounds ranging from 0.05 µg/mL to 10 µg/mL. Six different concentrations include 0.05 µg/mL, 0.1 µg/mL 0.5 µg/mL, 1 µg/mL, 5 µg/mL and 10 µg/mL were used to study linearity. The percentage drug entrapment was calculated as follows:

Percentage drug entrapment = [(Mass of total drug − Mass of free drug)/Mass of total drug] × 100

### 4.5. In Vitro Release Study

In vitro release studies of periglaucine A and betulinic acid loaded on to PLGA NPs were carried out in phosphate buffer solution (PBS) at pH 7.4. 15 mg of PGA-PLGA and BA-PLGA NPs. PGA-PLGA and BA-PLGA nanoparticles were suspended in 40 mL of PBS with continuous stirring for 72 h. At certain intervals, release medium was collected and replaced with fresh medium. The collected media was centrifuged and amount of periglaucine A and betulinic acid released were determined by UV-visible spectrophotometer at 630 nm and 210 nm.

### 4.6. Acanthamoeba Triangularis

#### 4.6.1. Acanthamoeba Cultivation Isolation and Cultivation

Environmental water samples were supplied by Department of Parasitology, Faculty of Medicine, University of Malaya. Water samples were cultivated on non-nutrient agar plates (NNA) lawned with *Escherichia coli* and incubated at 26 °C. The presence of *Acanthamoeba triangularis* trophozoites was confirmed 48 to 72 h after inoculation on NNA medium using an inverted microscope. Trophozoites were observed as moving cells with prominent vacuole and acanthapodias while cysts observed are triangular (3–5 arms) dominant double-walled structures. The homogenous culture of *Acanthamoeba triangularis* was obtained by continuous sub-cultures prior to DNA extraction of the parasite [22].

#### 4.6.2. Amoebicidal Activity of PGA-PLGA and BA-PLGA NPs

The trophocidal and cysticidal activities PGA-PLGA and BA-PLGA NPs at different concentrations were observed by trypan blue exclusion method as previously described [22]. A total of 200 μL of calibrated cysts and trophozoites were mixed with 200 μL of PGA-PLGA and BA-PLGA NPs at various concentrations ranging from 25 μg/mL to 100 μg/mL and inhibition of cell viability was calculated after 24, 48 and 72 h respectively.

### 4.7. Cytotoxicity Studies

The cytotoxicity of PGA-PLGA and BA-PLGA nanoparticles was studied using MTT assay [23]. The cytotoxicity profile of periglaucine A, PLGA NPs (without compound), PGA-PLGA and BA-PLGA NPs were studied. Six different concentrations of each sample ranging from 3.25 µg/mL to 200 µg/mL were tested against MRC-5 cell lines (Epithelial lung cells). MRC-5 cells were grown in Roswell Park Memorial Institute (RPMI) with 10% Fetal Bovine Serum (FBS). Five to six passages were done. Cultures were checked and media was changed after 48–72 h depending on the growth. On each passage, cells were seeded out into 25 cm^3^ flasks, Nunc (Roskilde, Denmark) at a concentration of 2.5 × 10^5^ cells/mL. Cells were seeded in 96-well plates at a density of 10^6^ cells/well and incubated at 37 °C in a CO_2_ incubator with 5% CO_2_ for 24 h before the addition of the tested compounds. After the addition of compounds and prepared nanoparticles, the plates were further incubated for another 24 h before proceeding. The MTT, Roche Diagnostics GmbH (Mannheim, Germany) assays were performed according to the Mosmann assay [23]. In MTT assays, the respiring cells (live cells) reduce the yellow tetrazolium salt to purple formazan crystals by dehydrogenase enzymes secreted by the mitochondria of metabolically active cells. Thus, the amount of formazan crystals formed is proportional to the number of viable cells. The absorbance was taken at 570 nm, SpectraMax M3, Multi-Mode Microplate reader, Molecular Devices (Washington, DC, USA). Cells without adding test reagents were taken as untreated cells with 100% viability and cells with RPMI 1640 media only were used as a blank.

The percent viability was calculated using the formula:

Percent viability = [(Abs_sample_ − Abs_blank_)/Abs_untreated_ − Abs_blank_] × 100

Therapeutic index (TI) was calculated as:

TI = CC_50_ of human lung epithelial cell line/IC_50_ of *Acanthamoeba triangularis*

### 4.8. Mechanism of Action

The terminal deoxynucleotidyltransferase-mediated dUTP-biotin nick end labeling, apoptotic detection kit, S7100, Millipore (Burlington, MA, USA) was used in the assay according to the manufacturer’s recommendation. Briefly the experiment was carried out in 6 well plate. Trophic cell stages were cultured and adjusted to a concentration of 10^6^ cells/mL and mixed with different concentrations of PGA-PLGA and BA-PLGA NPs and left to incubate for 24 h. Cells without added NPs were used as a control. After incubation, cells were fixed on silanized slides with 1% paraformaldehyde prior to TUNEL assay. All steps were performed following manufacturer protocols. Apoptotic cells will form a brown precipitate by binding terminal deoxynucleotidyl transferase (TdT) and conjugate drug and peroxidase, converting the substrate, a mixture of hydrogen peroxide and 3’3- diaminobenzidine (DAB) in a brown precipitate. The precipitate can be detected by light microscopy, identifying apoptotic nuclei with a dark brown color.

## 5. Conclusions

PLGA NPs were prepared and effectively loaded with periglaucine A and betulinic acid. The compounds effective release from PGA-PLGA and BA-PLGA NPs were studied. This pioneer study demonstrates the successful synthesis of PLGA nanoparticles encapsulated with periglaucine A and betulinic acid as well as its potent activity against pathogenic *Acanthamoeba triangularis*. These PGA-PLGA nanoparticles could provide a basis for better drug delivery systems by using these submicron size particles to make target-specific drugs and may be developed as novel and promising anti-*Acanthamoeba* agents for clinical use.

## Figures and Tables

**Table 1:** Effect of PGA-PLGA NPs on growth inhibition/stimulation of *Acanthamoeba triangularis*.

PGA-PLGA Dose (µg/mL)	Duration of Assay (Hours)					
	24 h		48 h		72 h	
Trophozoites	Mean ± SD	Growth Inhibition or Stimulation (%)	Mean ± SD	Growth Inhibition or Stimulation (%)	Mean ± SD	Growth Inhibition or Stimulation (%)
Non-treated control	18.0 ± 1.0	0	15.7 ± 1.2	0	13.3 ± 2.9	0
Chlorhexidine 0.004%	11.0 ± 1.0	38.8 (-)	10.0 ± 2.0	36.3 (-)	6.7 ± 0.6	49.9 (-)
BNP	18.0 ± 2.0	0	15.0 ± 1.0	0	14.0 ± 1.0	0
PGA-PLGA 100 µg/mL	7.7 ± 0.6 *	57.4 (-)	4.7 ± 0.6 *^,^**	70.3 (-)	3.3 ± 0.6 *^,^**	74.9 (-)
PGA-PLGA 50 µg/mL	11.3 ± 1.2 *	37.0 (-)	7.0 ± 1.0 *^,^**	55.4 (-)	5.3 ± 0.6 *	59.9 (-)
PGA-PLGA 25 µg/mL	8.3 ± 0.6 *	53.7 (-)	7.7 ± 0.6 *^,^**	50.6 (-)	6.7 ± 0.6	49.9 (-)
Cysts						
Non-treated control	32.0 ± 5.2	0	23.6 ± 7.0	0	19.0 ± 7.0	0
Chlorhexidine 0.025%	16.6 ± 7.2	47.9 (-)	14.0 ± 1.0	40.9 (-)	6.6 ± 2.5	49.1 (-)
BNP	32.0 ± 2.0	0	24.2 ± 4.0	0	19.0 ± 5.0	0
PGA-PLGA 100 µg/mL	22.6 ± 5.5 *	29.1 (-)	16.0 ± 1.7 *	32.5 (-)	11.0 ± 3.0 *	42.1 (-)
PGA-PLGA 50 µg/mL	15.6 ± 2.0 *	51.0 (-)	19.0 ± 1.0 *	19.3 (-)	14.3 ± 2.3 *	24.5 (-)
PGA-PLGA 25 µg/mL	26.6 ± 1.5 *	16.7 (-)	22.3 ± 1.5 *	9.3 (-)	19.0 ± 1.0	2.6 (-)

(+)—stimulation, (-)—inhibition. * *p* < 0.05, statistically significant difference in comparison to non-treated control in the same time interval. ** *p* < 0.05, statistically significant difference in comparison to drug control in the same time interval. BNP: Blank PLGA Nanoparticles.

**Table 2:** Effect of BA-PLGA NPs on growth inhibition/stimulation of *Acanthamoeba triangularis*.

BA-PLGA Dose (µg/mL)	Duration of Assay (Hours)					
	24 h		48 h		72 h	
Trophozoites	Mean ± SD	Growth Inhibition or Stimulation (%)	Mean ± SD	Growth Inhibition or Stimulation (%)	Mean ± SD	Growth Inhibition or Stimulation (%)
Non-treated control	22.6 ± 1.0	0	23.0 ± 1.2	0	22.3 ± 2.9	0
Chlorhexidine 0.004%	5.35 ± 1.1	76.4 (-)	14.0 ± 2.0	39.1 (-)	12.0 ± 0.5	46.1 (-)
BNP	22.0 ± 1.0	0	24.0 ± 1.0	0	22.5 ± 2.0	0
BA-PLGA 100 µg/mL	10.3 ± 0.6 *^,^**	45.5 (-)	15.6 ± 0.6 *	68.1 (-)	19.6 ± 0.6 *	71.3 (-)
BA-PLGA 50 µg/mL	15.6 ± 0.6 *^,^**	69.1 (-)	12.6 ± 0.6 *	55.0 (-)	11.6 ± 0.0 *	52.2 (-)
BA-PLGA 25 µg/mL	15.6 ± 0.6 *^,^**	69.1 (-)	15.6 ± 0.6 *	68.1 (-)	16.3 ± 1.6 *	88.0 (-)
Cysts						
Non-treated control	22.0 ± 5.2	0	23.6 ± 7.0	0	19.0 ± 7.0	0
Chlorhexidine 0.025%	16.6 ± 7.2	27.2 (-)	14.0 ± 1.0	39.1 (-)	12.0 ± 0.8	45.4 (-)
BNP	22.0 ± 4.0	0	23.0 ± 2.0	0	19.0 ± 3.0	0
BA-PLGA 100 µg/mL	10.3 ± 1.0 *^,^**	54.4 (-)	15.6 ± 3.7 *	38.8 (-)	16.3 ± 3.4 *	26.8 (-)
BA-PLGA 50 µg/mL	15.6 ± 3.6 *	30.8 (-)	12.6 ± 2.0 *	44.9 (-)	11.6 ± 4.0 *	47.6 (-)
BA-PLGA 25 µg/mL	15.6 ± 1.6 *	30.8 (-)	15.6 ± 1.6 *	31.8 (-)	19.6 ± 2.6 *	11.9 (-)

(+)—stimulation, (-)—inhibition. * *p* < 0.05, statistically significant difference in comparison to non-treated control in the same time interval. ** *p* < 0.05, statistically significant difference in comparison to drug control in the same time interval. BNP: Blank PLGA Nanoparticles.

**Table 3:** Cytotoxicity Tests.

S.No	Polymeric Nanoparticles	CC_50_ (µg/mL)		IC_50_ (µg/mL)	Therapeutic Index (TI)IC_50_ (µg/mL)/ CC_50_ (µg/mL)	
		Trophozoites	Cysts		Trophozoites	Cysts
1-	PLGA-PGA Nanoparticles	50	200	2	0.04	0.01
2-	BA-PLGA Nanoparticles	100	200	20	0.2	0.1
